# Laparoscopic Endoscopic Cooperative Surgery for Multiple Gastrointestinal Stromal Tumors Associated with Incomplete Carney Triad: A Case Report

**DOI:** 10.70352/scrj.cr.25-0222

**Published:** 2025-10-10

**Authors:** Ryuji Takada, Yuji Ishibashi, Akihiro Shimotakahara, Shin Namiki, Noriyuki Saito, Ryuichiro Furuta, Haruka Okada, Fumihiko Hatao, Kazuhiro Imamura, Yasuhiro Morita

**Affiliations:** 1 Department of Surgery, Tokyo Metropolitan Tama Medical Center, Fuchu, Tokyo, Japan; 2 Department of Surgery, Tokyo Metropolitan Children’s Medical Center, Fuchu, Tokyo, Japan; 3 Department of Gastroenterology, Tokyo Metropolitan Tama Medical Center, Fuchu, Tokyo, Japan; 4 Department of Pathology, Tokyo Metropolitan Tama Medical Center, Fuchu, Tokyo, Japan

**Keywords:** Carney triad, gastrointestinal stromal tumor, laparoscopic endoscopic cooperative surgery

## Abstract

**INTRODUCTION:**

Gastrointestinal stromal tumor (GIST) associated with Carney triad differs clinically, pathologically, and behaviorally from sporadic GIST. Surgical resection is the main form of treatment.

**CASE PRESENTATION:**

A 15-year-old female patient with an unremarkable medical history and family history was hospitalized for COVID-19. A chest X-ray incidentally revealed a pulmonary, nodular shadow. CT revealed a 55-mm pulmonary nodule with coarse calcification in the left lower lobe and gastric tumors in the upper body, on the basis of which pulmonary chondroma was diagnosed. Upper gastrointestinal endoscopy revealed 3 tumors, including a 10-mm tumor in the fornix and a 10- and 30-mm tumor in the upper gastric body. An endoscopic ultrasonography-guided fine needle biopsy revealed multiple GIST. On the basis of these findings, incomplete Carney triad was diagnosed. A partial gastrectomy via laparoscopic endoscopic cooperative surgery was performed for the GIST. Laparoscopy-assisted endoscopic full-thickness resection was performed first for the tumor in the fornix. Then, the 2 tumors in the upper gastric body were resected via a combination of laparoscopic and endoscopic approaches for neoplasia with non-exposure technique. The postoperative course was uneventful, and the patient was discharged on POD 11. Three months after the partial gastrectomy, a thoracoscopic left lower lobectomy was performed for the pulmonary chondroma. To date, 3 years after the surgery, there is no evidence of a recurrence.

**CONCLUSIONS:**

When resecting multiple GIST associated with Carney triad, surgeons should choose a surgical approach optimized for the individual patient and prioritize curability and preservation of the gastric function.

## Abbreviations

CD34
: cluster of differentiation 34
CLEAN-NET
: combination of laparoscopic and endoscopic approaches for neoplasia with non-exposure technique
GIST
: gastrointestinal stromal tumor
LAEFR
: laparoscopy-assisted endoscopic full-thickness resection
LECS
: laparoscopic endoscopic cooperative surgery
PDGFRA
: platelet-derived growth factor receptor A
SDHB
: succinate dehydrogenase B
SMA
: smooth muscle actin

## INTRODUCTION

Carney triad, first described in 1977, consists of the co-occurrence of multiple GIST, extra-adrenal paraganglioma, and pulmonary chondroma.^1)^ The presence of only 2 of these tumor types is known as incomplete Carny triad.^2)^ GIST associated with Carney triad differs clinically, pathologically, and behaviorally from sporadic GIST, and surgical resection is the main form of treatment.^3)^ Herein, we report a case of LECS for the excision of multiple GIST associated with incomplete Carney triad.

## CASE PRESENTATION

**Fig. 1:**
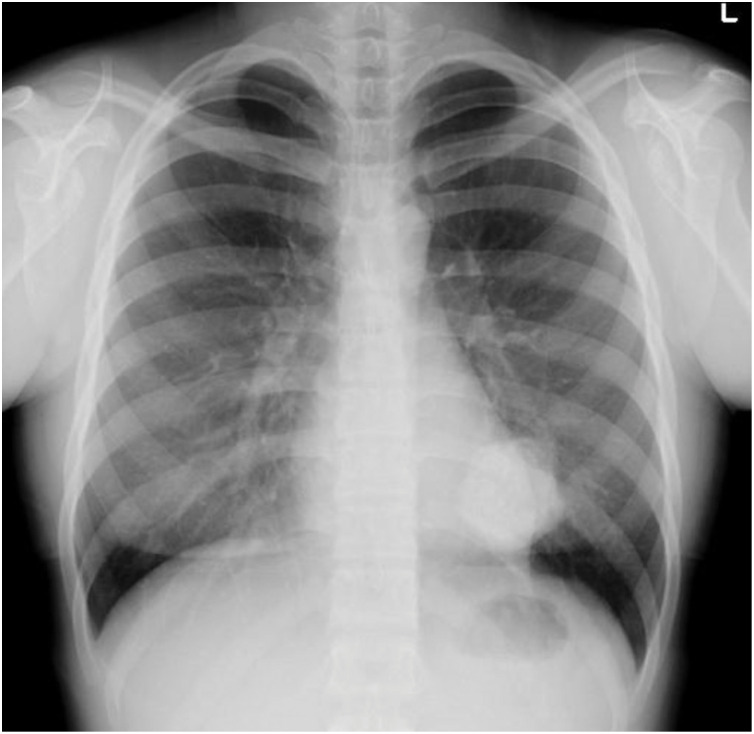
Chest X-ray.

**Fig. 2:**
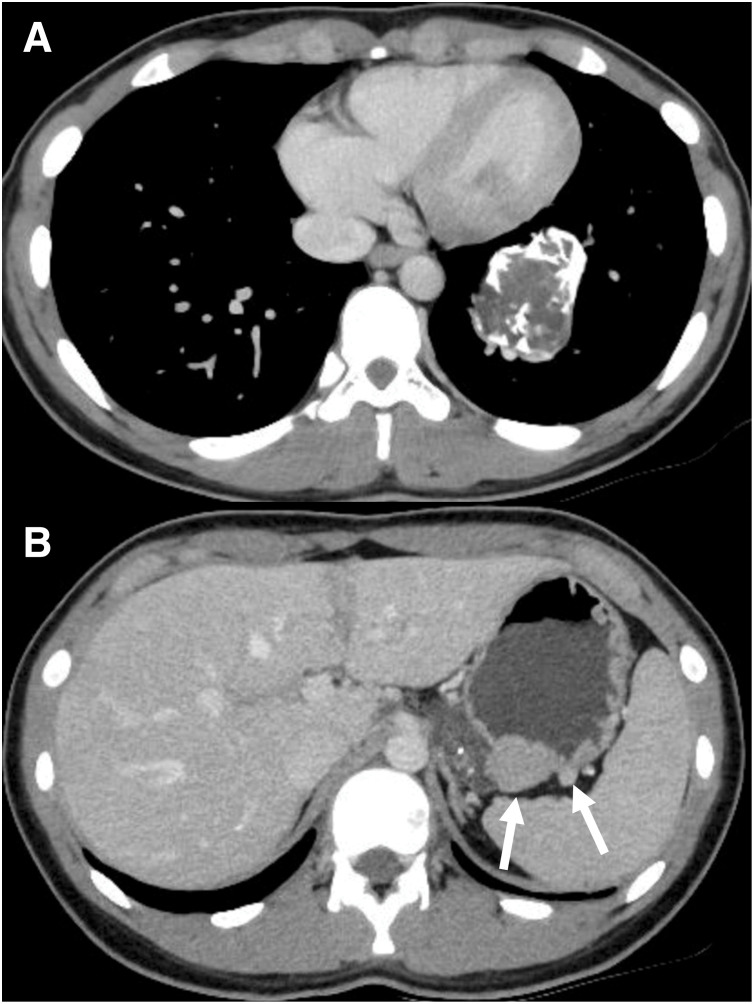
CT. (**A**) 55-mm pulmonary nodule with coarse calcification in the left lower lobe. (**B**) Gastric tumors in the upper body.

**Fig. 3:**
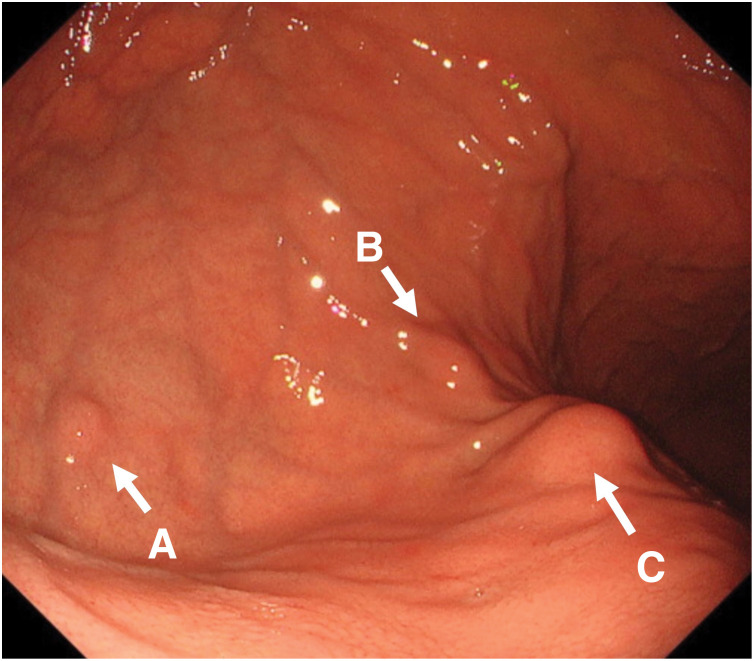
Upper gastrointestinal endoscopy. A 10-mm tumor (**A**) in the fornix and 10-mm (**B**) and 30-mm (**C**) tumor in the upper gastric body.

**Fig. 4:**
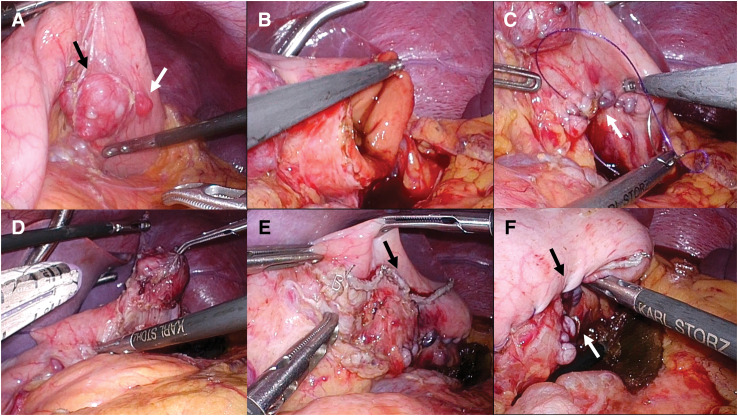
Intraoperative findings. (**A**) Tumor B (white arrow) and C (black arrow) were visible. (**B**) LAEFR was performed. (**C**) The gastric defect was closed by hand-sewing. The white arrow indicates the LAEFR suture line. (**D**) The seromuscular layer around tumors B and C was dissected, then the full layer was lifted. (**E**) The mucosa was transected with a stapler. The black arrow indicates the staple line. (**F**) The black arrow indicates the CLEAN-NET suture line, and the white arrow indicates the LAEFR suture line. LAEFR, laparoscopy-assisted endoscopic full-thickness resection; CLEAN-NET, combination of laparoscopic and endoscopic approaches for neoplasia with non-exposure technique

**Fig. 5:**
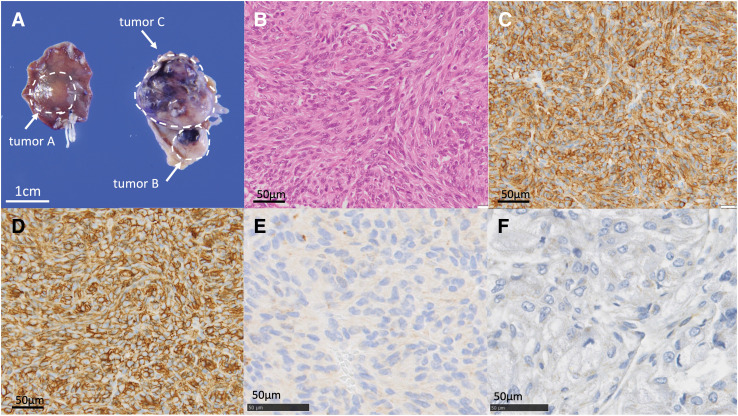
Pathological findings of GIST. (**A**) Macroscopic findings of a resection specimen. (**B**) Hematoxylin-eosin staining, ×400. (**C**) c-kit immunostain-positive. (**D**) CD34 immunostain-positive. (**E**) Loss of staining with PDGFRA immunostain. (**F**) Loss of staining with SDHB immunostain. CD34, cluster of differentiation 34; GIST, gastrointestinal stromal tumor; PDGFRA, platelet-derived growth factor receptor A; SDHB, succinate dehydrogenase B

**Fig. 6:**
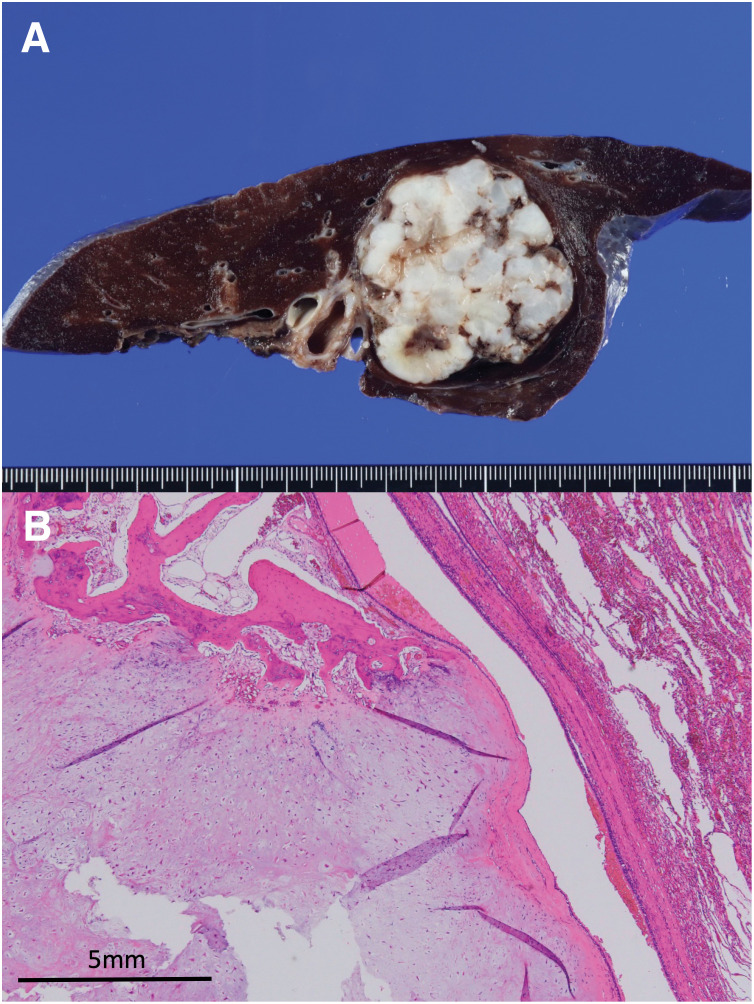
Pathological findings of the pulmonary chondroma. (**A**) Macroscopic findings of a resection specimen. (**B**) Hematoxylin-eosin staining, ×4. Proliferation of cartilage and bone tissue was observed. The tissue boundaries were surrounded by ciliated columnar epithelium.

A 15-year-old female patient with an unremarkable medical and family history was hospitalized for COVID-19. A chest X-ray incidentally revealed a pulmonary, nodular shadow (**Fig. 1**), prompting a referral to our hospital for further evaluation. CT revealed a 55-mm, pulmonary nodule with coarse calcification in the left lower lobe (**Fig. 2A**). Although a biopsy was not performed, pulmonary chondroma was diagnosed on the basis of the CT findings. While CT revealed gastric tumors in the upper body, upper gastrointestinal endoscopy revealed a 10-mm tumor in the fornix (tumor A) and 10- and 30-mm tumors in the upper gastric body (tumors B and C) (**Figs. 2B** and **3**). An endoscopic ultrasonography-guided fine needle biopsy revealed GIST in all the specimens. CT revealed a cystic tumor near the adrenal gland. MRI and 123I-metaiodobenzylguanidine scintigraphy revealed that the tumor was a lymphangioma rather than an extra-adrenal paraganglioma. Based on these findings, incomplete Carney triad was diagnosed. After obtaining consent for surgery from the patient and her parents, a partial gastrectomy via LECS was performed for the GIST (**Fig. 4**). After complete fundus and upper gastric body mobilization, the posterior stomach was inspected. Tumors B and C were visible in the upper gastric body, but tumor A in the fornix was not visible laparoscopically. No swollen lymph nodes were found around the stomach. LAEFR for tumor A was first performed to create a circumferential, submucosal incision around the tumor, while the deeper muscular and serosal layers were dissected endoscopically under laparoscopic guidance and the gastric defect was closed by hand-sewing. Then, tumors B and C, which were visible laparoscopically, were resected using a CLEAN-NET, that is, the seromuscular layer around tumors B and C was dissected, the full layer was lifted, and the mucosa was then transected with a stapler for a full-thickness resection of the tumors and the seromuscular defect was closed by hand-sewing. The total operative time was 308 minutes, and there was little blood loss. GIST was diagnosed on the basis of a pathological analysis of 8 × 8 × 8-mm, 11 × 10 × 9-mm and 22 × 22 × 20-mm nodules, which consisted of spindle cells with DOG-1(+), c-kit (+), CD34(+), S-100(−), and SMA(−). PDGFRA and SDHB were negative (**Fig. 5**). The Ki-67 labeling index was 4%. The postoperative course was uneventful. The patient was discharged on POD 11. Three months after the partial gastrectomy, a thoracoscopic left lower lobectomy was performed for the pulmonary nodule, and the patient was discharged on POD 6 without complications. Pathological findings demonstrated pulmonary chondroma (**Fig. 6**). Currently, at postoperative year 3, there is no evidence of a recurrence or change in the size of the lymphangioma near the adrenal grand.

## DISCUSSION

GIST is the most common mesenchymal tumor of the gastrointestinal tract. However, sporadic GIST differs from GIST associated with Carney triad; the latter has the distinctive features of female predilection, young age at onset, tumor multifocality, frequent lymph node metastases, serial tumor occurrences, unresponsiveness to imatinib, and unpredictable behavior.^4)^ Pathological findings of GIST associated with Carney triad include immunohistochemical negativity for SDHB expression and KIT or PDGFRA mutation, while most sporadic GIST is positive for SDHB expression and KIT or PDGFRA mutation.^5)^

The main treatment for GIST associated with Carney triad is surgical resection. Surgeons should decide on whether to perform a partial gastrectomy or systematic gastrectomy on the basis of the tumors’ multifocality, frequency of lymph node metastasis, and propensity to occur in young patients. Some reports have recommended performing a systematic gastrectomy with lymph node dissection because GIST associated with Carney triad frequently exhibit lymph node metastasis (47%) and multicentric occurrence.^4,6,7)^ However, since this condition mainly occurs in young patients, a partial gastrectomy should be considered with a view to preserving the gastric function. Midoritani et al.^8)^ reported a case of GIST associated with Carney triad for which laparoscopic total gastrectomy was performed. Initially they planned to perform a partial gastrectomy, but the intraoperative detection of lymph node metastasis required a total gastrectomy with lymph node dissection emphasizing curability. In the present case, after the patient’s age was taken into consideration, a partial gastrectomy was performed with the aim of preserving gastric function following confirmation of the absence of lymph node metastasis in preoperative and intraoperative examination findings. If lymph node metastasis is suspected, a systematic gastrectomy with lymph node dissection should promptly be performed with curative intent.

LECS, which combines endoscopic mucosal incision with laparoscopic surgery, is the chief partial gastrectomy technique for the treatment of submucosal tumors, including GIST. Hiki et al.^9)^ first reported the utility of classical LECS using adequate cut lines to excise gastric submucosal tumors safely. The major advantage of the LECS is that it requires less resection of the gastric wall than the conventional laparoscopic wedge resection using a linear stapler. LAEFR, described by Abe et al.,^10)^ and CLEAN-NET, described by Inoue et al.,^11)^ are both modifications of the classical LECS. In the present case, a partial gastrectomy was necessary to remove the 3 tumors without excessive gastric resection, which may lead to postoperative deformation of the stomach and gastric stasis. The mass in the fornix (tumor A) was an intra-luminal tumor and invisible from the serosal side; thus, LAEFR, which enables to create a circumferential, submucosal incision around the tumor under direct endoscopic visualization, was chosen for its resection. CLEAN-NET was performed for the 2 remaining, extra-luminal tumors in the upper gastric body (tumors B and C) because its procedure of cut-and-closing by linear stapling device can be completed using only a laparoscopic device and thus requires less time to complete than other techniques.

Long-term survival of GIST associated with Carney triad is generally favorable; Zhang et al.^4)^ reported a survival rate of 100% and 73% for postoperative 10 and 40 years, respectively.^12)^ However, GIST associated with Carney triad is aggressive, recurs frequently, and is prone to metastasis. Moreover, unlike sporadic GIST, they are resistant to imatinib because it has a different, pathophysiological mechanism. Patients with GIST associated with Carney triad are now categorized as high-risk and require regular, endoscopic and radiological surveillance for early detection of lymph node metastases, recurrence in the remnant stomach, and distant metastases. Grotz and Donohue has outlined recommendations for GIST surveillance: screening endoscopy should be performed every 3 years starting at the diagnosis; during postoperative years 1–3, history-taking, physical examinations, and CT every 3–4 months should be performed; then same procedures should be performed every 6 months until postoperative year 5, after which the procedures need to be performed only annually.^13)^ Fluorodeoxyglucose-PET is helpful in differentiating viable GIST from postoperative scar tissue or benign changes if CT findings are inconclusive. MRI may be helpful for detecting liver metastases. When performing a partial gastrectomy for GIST associated with Carney triad, as in the present case, surgeons should be aware that the tumor tends to recur in the remnant stomach. Zhang et al.^4)^ reported that of 81 patients who initially underwent segmental excision or a subtotal gastrectomy, 27 later required completion gastrectomy due to tumor development. Shimura et al.^7)^ reported a case of multiple GIST recurrences associated with incomplete Carny triad. The patient initially received a partial gastrectomy followed by a laparoscopic completion gastrectomy 11 years later. In our case, if the GIST were to recur in the remnant stomach, a total gastrectomy would be required owing to the probability of a recurrence in the remnant stomach if only a partial gastrectomy were performed again.

In our case, the pulmonary chondroma was resected after the partial gastrectomy. Pulmonary chondroma originating in the peribronchial mesenchyme occurs in 76% of patients with Carney triad,^12)^ where it is usually unilateral, solitary, and diagnosed incidentally, as in our patient. CT is useful for detecting peripheral calcification in the chondroma, which may not be evident on a standard radiograph, but the definitive diagnosis requires a biopsy. Even if the tumor’s biological behavior is benign, resection may be warranted given the potential of tumor to cause respiratory symptoms, recurrent pulmonary infections, or hemoptysis.^14)^ In our case, surgery was chosen to treat the tumor, which was large, measuring 55 mm in diameter, and considered likely to become symptomatic at a later stage.

While extra adrenal paraganglioma was not diagnosed in the present case, it was found to occur in 47% of patients with Carney triad.^12)^ Paraganglioma is a rare, neuroendocrine tumor originating in neural crest-derived chromaffin cells. Most paragangliomas associated with Carney triad are functional (hypertension, flushing, palpitations) and become locally invasive. Treatment consists of surgical resection, radiation, or embolization, depending on their resectability and the metastatic status of the lesion.^15)^

## CONCLUSIONS

For GIST associated with Carney triad, surgeons should choose a surgical method optimized for the individual patient while also prioritizing curability and gastric function.

## DECLARATIONS

### Availability of data and materials

All the data generated or analyzed during this study are included in this article.
